# Leukocyte-Based Inflammatory Profiles Across Dyslipidemia Phenotypes: Patterns of Eosinophil-Related Indices

**DOI:** 10.3390/medicina61091579

**Published:** 2025-08-31

**Authors:** Yazeed Alshuweishi, Muath Alsaidan, Ahmed M. Basudan, Hussam A. Aljohani, Hamad S. Almutairi, Nizar Algarni

**Affiliations:** 1 Department of Clinical Laboratory Sciences, College of Applied Medical Sciences, King Saud University, Riyadh 12372, Saudi Arabia; ahmbasudan@ksu.edu.sa (A.M.B.); halgohani@ksu.edu.sa (H.A.A.); 2 Department of Family and Community Medicine, College of Medicine, King Saud University, Riyadh 12372, Saudi Arabia; malsaidan@ksu.edu.sa; 3 Strategic Center for Diabetes Research, College of Medicine, King Saud University, Riyadh 11461, Saudi Arabia; 4 Department of Pathology and Laboratory Medicine, King Khalid University Hospital, King Saud University Medical City, Riyadh 12372, Saudi Arabia; hsalmutairi@ksu.edu.sa; 5 Department of Orthopedic Surgery, College of Medicine, King Saud University, Riyadh 12372, Saudi Arabia; anizar@ksu.edu.sa

**Keywords:** eosinophils, dyslipidemia, inflammation, ELR, EA-SIRI

## Abstract

*Background and Objectives:* Dyslipidemia, a modifiable cardiovascular risk factor, is associated with chronic low-grade inflammation. While leukocyte-derived indices have been investigated in this context, eosinophil-related inflammatory markers remain underexplored. This study examined patterns of eosinophil-to-lymphocyte ratio (ELR) and eosinophil-adjusted systemic inflammation response index (EA-SIRI) across dyslipidemia phenotypes. *Materials and Methods:* In this retrospective study, adult subjects were classified into six dyslipidemia phenotypes. Leukocyte-derived indices were evaluated across groups, and analyses included comparisons of medians, prevalence rates, tertile distributions, odds ratios, and risk estimates. *Results:* Both ELR and EA-SIRI were significantly higher in individuals with atherogenic dyslipidemia (ELR: 0.18; EA-SIRI: 1.53) and combined dyslipidemia (ELR: 0.17; EA-SIRI: 1.49) compared to the normolipidemic group (ELR: 0.11; EA-SIRI: 0.92). Notably, these patterns were more pronounced in males aged <40 years and younger females (<40), suggesting sex- and age-related variations in eosinophil-related inflammatory responses to dyslipidemia. Moreover, the highest tertiles of both ELR and EA-SIRI exhibited higher triglycerides and lower HDL-C compared to the lowest tertiles (*p* < 0.001). The odds of atherogenic dyslipidemia were more than doubled in individuals with elevated ELR (OR = 2.02; *p* < 0.001) and EA-SIRI (OR = 2.19; *p* < 0.001). ROC curve analysis indicated modest discriminative power for identifying atherogenic dyslipidemia, with ELR and EA-SIRI yielding AUC of 0.60 (*p* < 0.001) and 0.62 (*p* < 0.001), respectively. *Conclusions:* Our findings suggest eosinophil-related inflammation contributes to immunometabolic dysregulation underlying dyslipidemia. ELR and EA-SIRI may offer insights into inflammation-driven lipid disturbances and help detect subclinical inflammatory activity associated with atherogenic lipid profiles.

## 1. Introduction

Dyslipidemia (DLD) encompasses lipid abnormalities such as elevated total cholesterol (TC), low-density lipoprotein cholesterol (LDL-C), triglycerides (TG), or reduced high-density lipoprotein cholesterol (HDL-C) [1]. These changes may occur alone or in combination and represent a major modifiable risk factor for cardiovascular disease (CVD), the leading global cause of death [2]. Etiologies include genetic conditions, obesity, insulin resistance, and secondary disorders such as hypothyroidism and chronic kidney disease [3]. If left untreated, dyslipidemia promotes endothelial dysfunction, atherogenesis, and vascular inflammation, contributing to myocardial infarction and stroke [4]. DLD is also associated with metabolic syndrome, type 2 diabetes, nonalcoholic fatty liver disease, and systemic inflammation, particularly in profiles with low HDL-C and high TG [5,6,7]. According to an AHA scientific statement, lipid abnormalities remain a major driver of global CVD burden [8]. A key challenge is that DLD is typically asymptomatic and often begins early, with atherogenic lipid profiles emerging during adolescence or early adulthood, largely due to obesity and inactivity [9,10]. This underscores the importance of early screening and intervention to prevent long-term vascular and metabolic complications.

In Saudi Arabia, dyslipidemia has emerged as a major non-communicable health concern. It is highly prevalent across age groups where school-based and adolescent studies report early onset of lipid abnormalities, strongly influenced by obesity and lifestyle [11,12]. Among adults, a 2020 survey in Jeddah reported dyslipidemia in 62% of participants, with elevated LDL-C (40.9%), low HDL-C (24.4%), and hypertriglyceridemia (21.7%) being the most common abnormalities [13]. Dyslipidemia is also common in chronic conditions such as type 2 diabetes and metabolic syndrome [14]. In inflammatory disease cohorts, hyperlipidemia was observed in 11.4% of psoriatic arthritis patients, reflecting an added metabolic burden [15]. Collectively, these findings highlight the widespread and multifaceted nature of dyslipidemia across age groups and clinical populations in Saudi Arabia, emphasizing the need for early detection and targeted intervention.

A growing body of literature emphasizes the role of inflammation in dyslipidemia, with chronic low-grade inflammation recognized as a key factor in atherosclerosis and metabolic dysfunction [16,17]. Lipid species such as fatty acids, lipoproteins, and sphingolipids exert immunomodulatory effects, acting as pro- or anti-inflammatory agents and influencing cardiometabolic disease [18]. In turn, inflammatory mediators may disrupt lipid metabolism, creating a cycle of metabolic and inflammatory disturbances [19]. Consequently, this complex interplay between dyslipidemia and systemic inflammation has garnered increasing attention in both clinical and research contexts. In this context, hematologic biomarkers derived from routine complete blood counts (CBCs) have emerged as accessible and cost-effective tools for assessing systemic inflammation in metabolic and cardiovascular disorders [20]. Composite indices based on white blood cell counts—most notably the neutrophil-to-lymphocyte ratio (NLR), monocyte-to-lymphocyte ratio (MLR), and the systemic inflammation response index (SIRI)—have shown associations with adverse outcomes in type 2 diabetes, including mortality, retinopathy, and kidney disease [21,22,23]. These indices have shown significant associations with adverse clinical outcomes in individuals with type 2 diabetes, including increased mortality, diabetic retinopathy, and diabetic kidney disease [24,25,26]. Their appeal lies in their simplicity, reproducibility, and availability from standard laboratory tests, supporting their use in large-scale screening and risk stratification.

Despite growing interest in leukocyte-based inflammatory markers, eosinophils have received comparatively little attention in dyslipidemia. Traditionally associated with allergic responses and parasitic infections, eosinophils are now recognized to have broader immunoregulatory roles, including participation in adipose tissue remodeling, metabolic inflammation, and vascular dysfunction. Recent studies suggest eosinophil counts and eosinophil-derived ratios may be associated with cardiovascular pathology, including atherosclerosis and acute coronary syndromes [27,28]. However, the clinical relevance of eosinophil-based inflammatory indices such as the eosinophil-to-lymphocyte ratio (ELR) or eosinophil-adjusted SIRI (EA-SIRI) in dyslipidemia has not been well characterized. This study therefore investigated associations between eosinophil-derived indices and specific dyslipidemia phenotypes in a large adult population, comparing their performance with traditional markers (NLR, MLR) and examining variation by age and sex. Our aim was to clarify the immunoinflammatory profiles linked to dyslipidemia and to assess whether eosinophil-based indices may offer value as accessible biomarkers in routine clinical practice.

## 2. Materials and Methods

### 2.1. Data Collection and Study Design

This retrospective cross-sectional study was conducted using data obtained from Elta Medical Laboratory. Subjects were drawn from Elta Medical Laboratories and included both walk-in individuals undergoing routine health check-ups and patients referred by physicians. A total of 7782 subjects were initially screened. Inclusion criteria required complete data for both complete blood count (CBC) and lipid profiles. After applying these criteria, 6963 individuals were included in the final analysis. Based on their lipid profiles, participants were classified into the following dyslipidemia phenotypes: normolipidemic profile (NLP, *n* = 3338), isolated hypercholesterolemia (IHC, *n* = 2135), isolated hypertriglyceridemia (IHTG, *n* = 175), isolated low HDL-C (ILHDL, *n* = 735), atherogenic dyslipidemia (AD, *n* = 211), and combined dyslipidemia (CD, *n* = 370).

Dyslipidemia phenotypes were defined based on the criteria set by the National Cholesterol Education Program-Adult Treatment Panel III (NCEP-ATP III) and classified into four phenotypes [29]. Isolated hypercholesterolemia (IHC) was defined as elevated TC and/or LDL-C with normal TG and HDL-C. Isolated hypertriglyceridemia (IHTG) was defined as elevated TG with normal TC, LDL-C, and HDL-C. Isolated low HDL-C (ILHDL) was defined as reduced HDL-C with other lipids within normal range. Atherogenic dyslipidemia (AD) was specifically defined as the coexistence of elevated TG and reduced HDL-C without elevated LDL-C or TC. Combined dyslipidemia (CD) was defined by the coexistence of elevated LDL-C accompanied by high TG and low HDL-C. These subgroupings are consistent with the previous literature and reflect clinically relevant patterns of lipid disturbance associated with increased cardiometabolic risk [30,31].

The study protocol and design were reviewed and approved by the Institutional Review Board at King Saud University, Riyadh, Saudi Arabia (Approval No: E-25-9893). As the study utilized de-identified routine laboratory data, the requirement for informed consent was waived in accordance with institutional and ethical guidelines.

### 2.2. Statistical Analysis

The relationship between eosinophil-based inflammatory indices and various DLD phenotypes was analyzed using nonparametric statistical methods, as the D’Agostino–Pearson and Kolmogorov–Smirnov tests confirmed non-normal data distribution (*p* < 0.001). Data were summarized as medians with interquartile ranges (IQRs) for continuous variables and as frequencies and percentages for categorical variables. Group comparisons across dyslipidemia phenotypes were assessed using the Kruskal–Wallis test. Post hoc analyses were conducted with Dunn’s test, in which the mean rank of each dyslipidemia group was compared against the normolipidemic (control) group. *p*-values were adjusted for multiple comparisons using the Bonferroni correction. Correlations between eosinophil-related indices—ELR and EA-SIRI—and lipid variables or DLD phenotypes were assessed using Spearman’s rank correlation. Risk associations between elevated eosinophil-based indices and specific DLD phenotypes were evaluated by calculating prevalence ratios (PRs) and odds ratios (ORs). Receiver operating characteristic (ROC) curve analysis was applied to determine the discriminative ability of ELR and EA-SIRI for identifying high-risk dyslipidemic profiles, with diagnostic accuracy assessed via the area under the curve (AUC). Statistical analyses were performed using GraphPad Prism version 9.2.0 (GraphPad Software, Inc., San Diego, CA, USA), with a significance threshold set at *p* < 0.05.

## 3. Results

### 3.1. Baseline Characteristics of the Studied Population

Table 1 presents the clinical and hematological characteristics of the study population stratified by dyslipidemia phenotypes. Age and sex distribution were generally comparable, with only a slight underrepresentation of females in the AD and CD groups. In contrast, significant differences were observed in leukocyte profiles, with WBC, neutrophil, and eosinophil counts elevated in hypertriglyceridemic and combined dyslipidemia phenotypes. Eosinophil levels showed a progressive increase, peaking in AD and CD. Monocytes and lymphocytes were also higher in dyslipidemic groups compared to normolipidemic individuals. Among red cell parameters, Hb and RBC were modestly higher in CD and AD, whereas platelet counts were lower. CRP and fasting glucose differed significantly across phenotypes, with higher median FBG levels noted in IHC, ILHDL, and AD.

### 3.2. Patterns of Leukocyte-Derived Inflammatory Ratios Across Dyslipidemia Phenotypes

**Figure 1:**
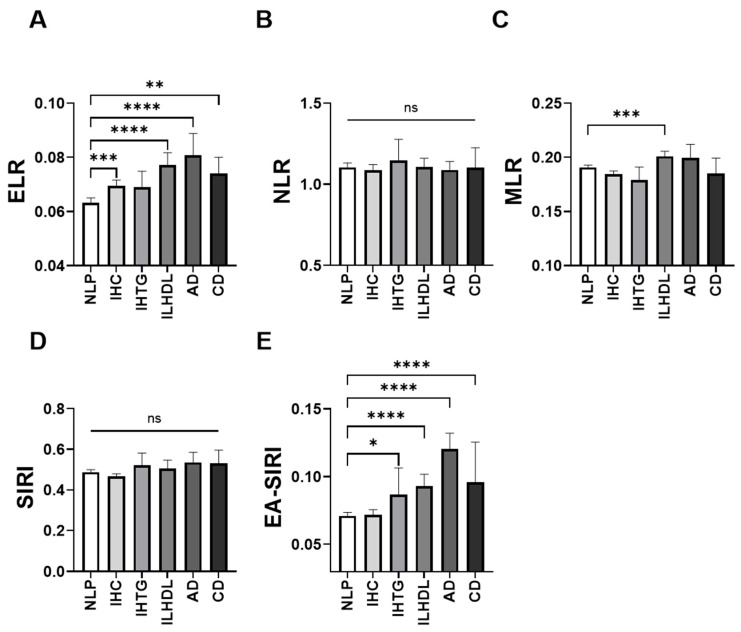
Comparison of leukocyte-derived inflammatory indices across dyslipidemia phenotypes. Bar plots depict the median levels of five leukocyte-based inflammatory markers, including (**A**) eosinophil-to-lymphocyte ratio (ELR), (**B**) neutrophil-to-lymphocyte ratio (NLR), (**C**) monocyte-to-lymphocyte ratio (MLR), (**D**) systemic inflammation response index (SIRI), and (**E**) eosinophil-adjusted systemic inflammation response index (EA-SIRI)*,* across six lipid profile categories: normolipidemic profile (NLP), isolated hypercholesterolemia (IHC), isolated hypertriglyceridemia (IHTG), isolated low high-density lipoprotein cholesterol (ILHDL), atherogenic dyslipidemia (AD), and combined dyslipidemia (CD). ns, not significant; * *p* < 0.05; ** *p* < 0.01; *** *p* < 0.001; **** *p* < 0.0001.

A comparative analysis of leukocyte-derived inflammatory indices across dyslipidemia phenotypes revealed distinct patterns, particularly in ratios involving eosinophils and monocytes. In Figure 1A, ELR showed a progressive elevation in dyslipidemic phenotypes, with significantly higher median values in ILHDL [0.077 (±0.047–0.123)], AD [0.081 (±0.055–0.113)], and CD [0.074 (±0.050–0.115)] compared to NLP [0.063 (±0.042–0.101)]. In contrast, NLR remained unchanged across groups with no significant differences (Figure 1B). In Figure 1C, MLR was modestly but significantly higher in ILHDL [0.201 (±0.164–0.247)] and IHC [0.185 (±0.153–0.228)] compared to NLP [0.191 (±0.156–0.236)]. Moreover, SIRI showed a nonsignificant trend toward higher values in AD and CD (Figure 1D). By contrast, EA-SIRI demonstrated a consistent increase (Figure 1E), with significantly higher medians in IHTG [0.087 (±0.041–0.182)], ILHDL [0.093 (±0.044–0.193)], AD [0.121 (±0.058–0.195)], and CD [0.096 (±0.051–0.198)] versus NLP [0.071 (±0.036–0.135)] (all *p* < 0.05).

### 3.3. Sex- and Age-Stratified Differences in Eosinophil-to-Lymphocyte Ratio (ELR) Across Dyslipidemia Phenotypes

**Figure 2:**
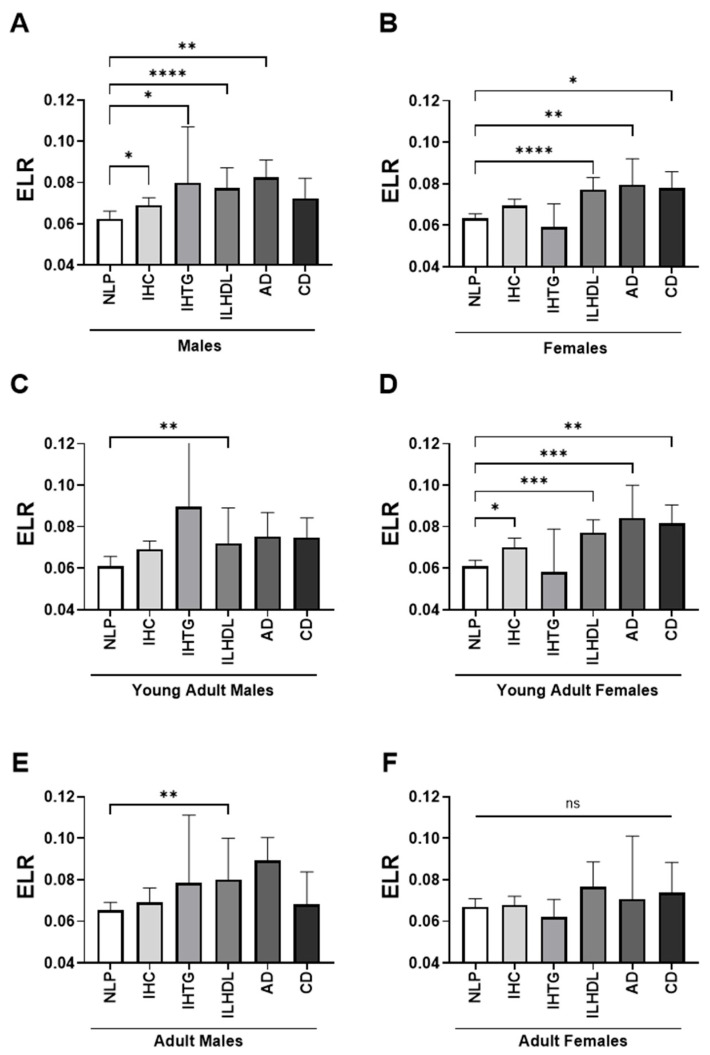
Age- and sex-stratified distribution of eosinophil-to-lymphocyte ratio (ELR) across dyslipidemia phenotypes. Bar plots depict the median levels of ELR across different dyslipidemia phenotypes, stratified by sex and age group. (**A**) Males (overall), (**B**) Females (overall), (**C**) Young adult males (<45 years), (**D**) Young adult females (<45 years), (**E**) Adult males (≥45 years), (**F**) Adult females (≥45 years). The six dyslipidemia phenotype groups are displayed from left to right in each plot as follows: normolipidemic profile (NLP), isolated hypercholesterolemia (IHC), isolated hypertriglyceridemia (IHTG), isolated low high-density lipoprotein cholesterol (ILHDL), atherogenic dyslipidemia (AD), and combined dyslipidemia (CD). ns, not significant; * *p* < 0.05; ** *p* < 0.01; *** *p* < 0.001; **** *p* < 0.0001.

Sex- and age-specific analysis revealed distinct patterns of ELR variation across dyslipidemia phenotypes. Among male participants, ELR was significantly higher in IHC [0.069 (±0.046–0.107)], IHTG [0.080 (±0.050–0.107)], ILHDL [0.077 (±0.047–0.123)], and AD [0.082 (±0.056–0.113)] compared to the normolipidemic group [0.063 (±0.042–0.101)], while no significant difference was observed for CD (Figure 2A). This trend was particularly evident in young adult males (≤40 years), where ILHDL showed the highest median ELR [0.072 (±0.048–0.119)] with a significant difference versus NLP (Figure 2C). In older males (>40 years), the highest ELR was observed in AD [0.089 (±0.055–0.113)], but only ILHDL remained significantly different from NLP (Figure 2E).

Among females, ELR was also elevated in dyslipidemic groups, with significant differences in ILHDL [0.077 (±0.047–0.121)], AD [0.080 (±0.054–0.112)], and CD [0.078 (±0.048–0.113)] compared to NLP [0.063 (±0.041–0.103)] (Figure 2B). In young adult females, ELR increased significantly in IHC [0.070 (±0.043–0.107)], ILHDL [0.077 (±0.046–0.122)], AD [0.084 (±0.056–0.116)], and CD [0.082 (±0.050–0.116)] relative to NLP [0.061 (±0.040–0.101)] (Figure 2D). However, in older adult females (>40 years), no significant differences were observed across phenotypes despite numerical trends toward higher ELR values (Figure 2F).

### 3.4. Sex- and Age-Stratified Differences in EA-SIRI Across Dyslipidemia Phenotypes

**Figure 3:**
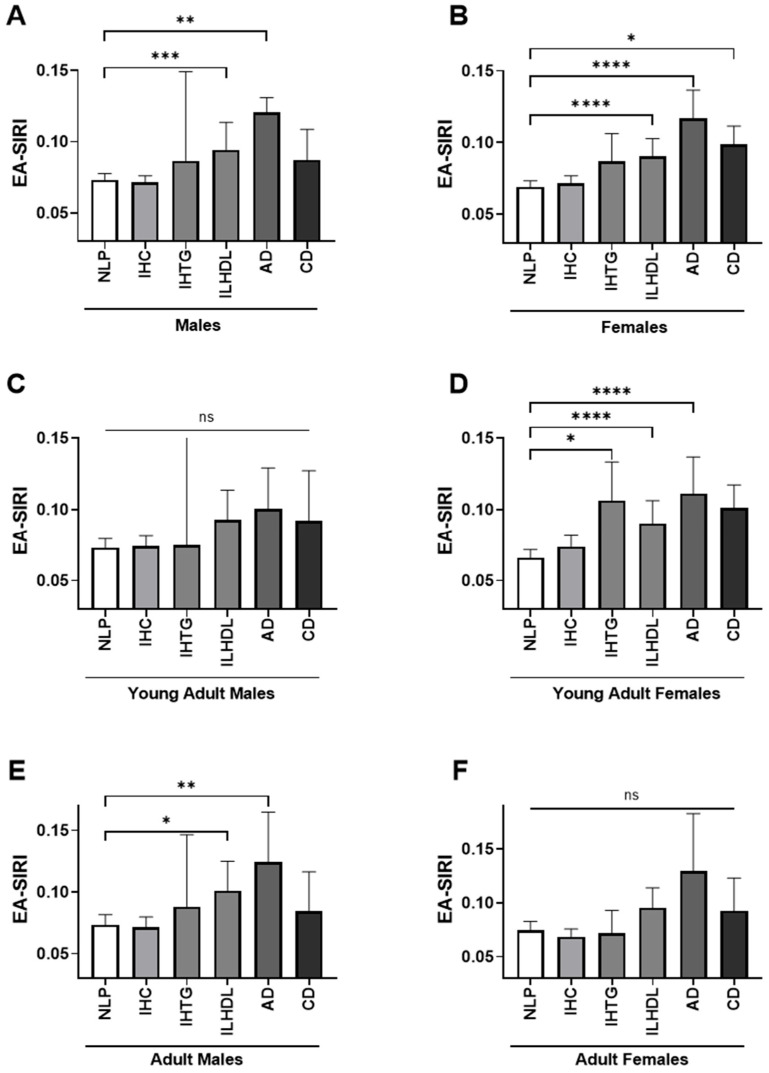
Age- and sex-stratified distribution of eosinophil-adjusted systemic inflammation response index (EA-SIRI) across dyslipidemia phenotypes. Bar plots depict the median levels of the EA-SIRI across different dyslipidemia phenotypes, stratified by sex and age group. (**A**) Males (overall), (**B**) Females (overall), (**C**) Young adult males (<45 years), (**D**) Young adult females (<45 years), (**E**) Adult males (≥45 years), (**F**) Adult females (≥45 years). The six dyslipidemia phenotype groups are displayed from left to right in each plot as follows: normolipidemic profile (NLP), isolated hypercholesterolemia (IHC), isolated hypertriglyceridemia (IHTG), isolated low high-density lipoprotein cholesterol (ILHDL), atherogenic dyslipidemia (AD), and combined dyslipidemia (CD). ns, not significant; * *p* < 0.05; ** *p* < 0.01; *** *p* < 0.001; **** *p* < 0.0001.

Analysis of the EA-SIRI revealed sex- and age-specific differences across dyslipidemia phenotypes. Among males, EA-SIRI was significantly elevated in individuals with ILHDL [0.094 (±0.043–0.209)] and AD [0.121 (±0.057–0.189)] compared to normolipidemic males [0.073 (±0.037–0.140)] (Figure 3A). These associations were more pronounced in older adult males (>40 years), where EA-SIRI reached 0.101 (±0.037–0.228) in ILHDL and 0.124 (±0.065–0.217) in AD, both significantly higher than NLP [0.073 (±0.036–0.142)] (Figure 3E). In contrast, no significant differences were observed among younger adult males (≤40 years), although EA-SIRI values were numerically elevated in ILHDL, AD, and CD (Figure 3C).

In females, EA-SIRI also demonstrated a consistent rise in dyslipidemic phenotypes, particularly those associated with low HDL-C and mixed lipid abnormalities. Median EA-SIRI was significantly higher in ILHDL [0.091 (±0.045–0.184)], AD [0.117 (±0.059–0.198)], and CD [0.099 (±0.045–0.152)] compared to normolipidemic females [0.069 (±0.036–0.133)] (Figure 3B). Among young adult females, this elevation persisted, with significant differences observed in IHTG [0.106 (±0.044–0.232)], ILHDL [0.090 (±0.045–0.191)], and AD [0.111 (±0.065–0.197)] versus NLP [0.066 (±0.034–0.130)] (Figure 3D). However, no significant differences in EA-SIRI were observed across dyslipidemia phenotypes in older adult females (>40 years), despite slightly higher values in AD and ILHDL (Figure 3F).

### 3.5. ELR Stratification Reveals Trends in Lipid Abnormalities and Dyslipidemia Types

Analysis of lipid profiles across ELR tertiles (Table 2) revealed a consistent trend toward adverse lipid parameters with rising ELR. Median TG increased from 83 mg/dL in T1 to 94 mg/dL in T3 (*p* < 0.001), while HDL-C declined from 50 mg/dL to 47 mg/dL (*p* < 0.001). TC and LDL-C also rose modestly but significantly (*p* = 0.003 and *p* = 0.001). Dyslipidemia phenotype distribution shifted accordingly: the proportion of normolipidemic individuals decreased from 52.91% in T1 to 43.86% in T3, while ILHDL increased from 9.13% to 13.31%. Atherogenic phenotypes also became more common, with AD rising from 1.85% to 3.83% and CD from 4.52% to 5.82%. These findings demonstrate that higher ELR is linked to progressively unfavorable lipid profiles, particularly those defined by elevated TG and reduced HDL-C.

### 3.6. Distribution of Lipid Parameters and Dyslipidemia Phenotypes by EA-SIRI Stratification

Table 3 shows that higher EA-SIRI tertiles were associated with progressively adverse lipid profiles. Median TG increased from 82 mg/dL in T1 to 96 mg/dL in T3 (*p* < 0.001), while HDL-C declined from 50 mg/dL to 47 mg/dL (*p* < 0.001). TC and LDL-C remained stable, with no significant differences (*p* = 0.5154 and *p* = 0.209), suggesting that EA-SIRI may be more reflective of TG- and HDL-C–related disturbances than of overall cholesterol burden. Dyslipidemia phenotype distribution followed a similar pattern. Normolipidemic prevalence declined from 51.27% to 43.52%, while ILHDL rose from 8.79% to 12.97%, AD from 1.81% to 4.61%, and CD from 4.44% to 6.68% across tertiles.

### 3.7. Frequency Distribution of Dyslipidemia Phenotypes in Relation to Elevated ELR and EA-SIRI Levels

The distribution of dyslipidemia phenotypes in relation to ELR and EA-SIRI stratification is presented in Table 4, revealing distinct patterns linked to inflammatory burden. Individuals with high ELR levels exhibited a notable reduction in the frequency of IHC, decreasing from 51.52% in the low ELR group to 43.52% in the high ELR group. Similarly, IHC prevalence also declined sharply with elevated EA-SIRI, from 49.99% in the low group to 35.32% in the high group, indicating a negative association between these inflammatory indices and isolated hypercholesterolemia.

Conversely, phenotypes associated with AD showed an upward trend with increasing ELR and EA-SIRI levels. The proportion of AD increased from 9.02% to 12.44% in the high ELR group and from 8.97% to 9.81% in the high EA-SIRI group. CD also became more prevalent with elevated inflammation, rising from 2.29% to 3.94% in the ELR groups and from 2.22% to 3.17% across EA-SIRI strata. Interestingly, IHTG exhibited a slight increase with higher ELR but a marked decrease with elevated EA-SIRI, suggesting divergent inflammatory associations depending on the index used. The frequency of ILHDL remained largely stable across both ELR and EA-SIRI groups.

### 3.8. Risk Estimates of Dyslipidemia Phenotypes According to Elevated ELR and EA-SIRI Levels

The risk assessment of dyslipidemia phenotypes based on elevated ELR and EA-SIRI levels (Table 5) revealed distinct patterns of association. Elevated ELR was significantly associated with increased odds of several dyslipidemia phenotypes, including ILHDL (OR: 1.55, 95% CI: 1.32–1.82, *p* < 0.001), AD (OR: 2.02, 95% CI: 1.52–2.68, *p* < 0.001), and CD (OR: 1.49, 95% CI: 1.20–1.85, *p* = 0.0003). Elevated ELR was also significantly associated with IHTG (OR: 1.40, 95% CI: 1.03–1.90, *p* = 0.030), whereas no significant association was observed with IHC (OR: 0.999, *p* = 0.9858). Similarly, elevated EA-SIRI was strongly associated with ILHDL (OR: 1.63, 95% CI: 1.39–1.92, *p* < 0.001), AD (OR: 2.04, 95% CI: 1.54–2.71, *p* < 0.001), and CD (OR: 1.51, 95% CI: 1.22–1.87, *p* = 0.0002). Additionally, EA-SIRI was positively associated with IHC (OR: 1.25, 95% CI: 1.12–1.40, *p* = 0.001), but not with IHTG (OR: 1.12, *p* = 0.4667).

### 3.9. Discriminatory Performance of ELR and EA-SIRI in Identifying Dyslipidemia Phenotypes

ROC analysis (Table 6) showed that ELR had modest discriminatory capacity, with the highest AUC for AD (AUC: 0.6002), followed by ILHDL (AUC: 0.5721) and CD (AUC: 0.5695). ELR showed little utility for IHTG (AUC: 0.5228) or IHC (AUC: 0.5302). EA-SIRI demonstrated slightly stronger performance, particularly for AD (AUC: 0.6202) and CD (AUC: 0.6033). It performed similarly to ELR for ILHDL (AUC: 0.5739) but showed better detection of IHTG (AUC: 0.5624). As with ELR, it was not useful for IHC (AUC: 0.5076). For ELR, the Youden cut-off was >0.073, providing a sensitivity of 58.8% and a specificity of 58.7%. Similarly, the optimal Youden cut-off for EA-SIRI was >0.088, with a sensitivity of 58.8% and a specificity of 58.6%. These ROC-derived thresholds are summarized in Table 7.

## 4. Discussion

This study investigated the clinical relevance of leukocyte-based inflammatory indices, particularly ELR and EA-SIRI, in relation to dyslipidemia (DLD) phenotypes in a large adult population. This study demonstrated that eosinophil-based inflammatory indices, specifically ELR and EA-SIRI, were significantly elevated in individuals with atherogenic and combined dyslipidemia phenotypes, compared to normolipidemic individuals. Notably, these elevations were more pronounced in older adult males and younger adult females under 40 years of age, indicating potential sex- and age-related differences in eosinophil-associated inflammation. Tertile analysis further revealed that individuals in the highest tertiles of ELR and EA-SIRI had noticeably lower HDL-C and higher triglyceride levels than those in the lowest tertiles. Additionally, both indices showed the highest odds of association with the atherogenic dyslipidemia (AD) phenotype, suggesting a stronger inflammatory component in this subgroup. Receiver operating characteristic (ROC) curve analysis showed that ELR and EA-SIRI had modest discriminative performance in identifying individuals with AD, suggesting a possible supportive role as accessible inflammatory markers in dyslipidemia screening and risk stratification. Notably, both indices showed the strongest link to the dyslipidemia phenotypes and outperformed conventional markers such as NLR, MLR, and SIRI in identifying atherogenic lipid profiles.

In the present study, we observed distinct patterns in the associations of leukocyte-derived inflammatory indices with dyslipidemia phenotypes in a large Saudi population. MLR was selectively associated with the ILHDL phenotype, whereas NLR did not show significant variation across any dyslipidemia subtype. This finding aligns with previous work by Wang et al., who reported that NLR did not exhibit any significant correlation with the risk of metabolic diseases [32]. One possible explanation is that NLR predominantly reflects acute inflammatory responses, which may not adequately capture the chronic low-grade inflammation commonly accompanying dyslipidemia. However, unlike Wang et al.’s findings, the SIRI in our study was not significantly associated with any dyslipidemia phenotype. While the reasons for this discrepancy remain unclear, they may relate to differences in population characteristics, including ethnicity, lifestyle, comorbidities, and the clinical criteria used to define dyslipidemia. Additionally, variations in inflammatory profiles and immune cell dynamics across populations could also influence the diagnostic utility of SIRI in detecting lipid abnormalities.

Eosinophil-associated indices outperformed neutrophil- or monocyte-based ratios, showing significant associations with all dyslipidemia phenotypes except IHC phenotype. Associations were strongest for disturbances involving high TG or low HDL-C, suggesting that eosinophil-related activity may be linked to lipid disturbances. Population-based studies, however, report mixed findings. Cohorts from the Netherlands, Japan, and China linked elevated eosinophils with lower HDL-C and higher LDL-C, TG, and TC [33,34], while U.S. multi-ethnic data showed no associations [35], and a British cohort even reported inverse relationships [36] (see Supplementary Table S1 for a summary of population-based studies). Similarly, experimental data support both protective and pathogenic roles of eosinophils in the context of lipid metabolism. Eosinophil-derived IL-4 and IL-13 promote M2 macrophage polarization and adipocyte maturation, enhancing lipid storage and insulin sensitivity, whereas eosinophil deficiency leads to impaired adipogenesis, ectopic lipid deposition, and glucose intolerance [37,38]. Moreover, eosinophils express fatty acid-binding protein 4 (FABP4), linking lipid mediator flux to eosinophil activation [39]. In contrast, under metabolic stress, eosinophils may exert pro-atherogenic effects. Large-scale epidemiological studies show positive associations with TG, TC, and LDL-C, and inverse associations with HDL-C, with BMI modifying these relationships [40,41]. A non-linear inverted-U pattern with adiposity has also been observed [41]. Lipidomics of eosinophilic COPD exacerbations reveal enrichment of triglyceride-rich lipoproteins and lysophosphatidylcholine [42], while metabolomic studies report inverse links with key lipid intermediates [34]. Together, current evidence supports a dual role: protective in physiologic states but pro-atherogenic in obesity and dyslipidemia, contextualizing our finding that eosinophil-related indices associate with atherogenic lipid profiles.

Our findings indicate a sex- and age-dependent association between eosinophil-related indices, both ELR and EA-SIRI, and dyslipidemia, with significant associations observed in adult males over 40 years of age and younger adult females under 40. This pattern is consistent with evidence that sex and age influence eosinophil count and function, thereby affecting their relevance in metabolic conditions. Large-scale studies have shown that adult males generally exhibit higher baseline eosinophil counts than females, even after adjusting for atopic diseases and metabolic syndrome [43,44], suggesting possible biological differences in eosinophil regulation. Moreover, molecular profiling studies further reveal distinct sex-related gene expression patterns in eosinophil-associated pathways [45]. In males, eosinophil activity appears more strongly correlated within inflammatory and metabolic disease markers, whereas in females, alternative regulatory mechanisms may be more prominent [45]. Age is also a critical modifier of eosinophil biology as both eosinophil counts and immunomodulatory functions tend to decline with advancing age, potentially reducing their contribution to metabolic regulation and inflammatory control in older individuals [44]. Supporting this, Brigger et al. reported that eosinophil numbers and activity in adipose tissue decline with age, which may contribute to worsening metabolic disturbances and inflammation [46]. Taken together, these findings highlight the modifying role of sex and age on the observed associations between eosinophil-related indices and dyslipidemia. However, our dataset did not include information on menopausal status or lifestyle factors, which may further influence sex- and age-specific patterns. Menopause is characterized by declining estrogen, leading to increases in total cholesterol, LDL-C, and triglycerides, along with unfavorable shifts in HDL subfractions [47,48,49]. Lower sex hormone-binding globulin (SHBG) is also strongly associated with high TG and low HDL-C [50]. After age 50, women often exhibit more adverse lipid profiles than men [51], and the effects of androgens such as testosterone and DHEAS on dyslipidemia remain variable across studies [52]. Future studies incorporating these hormonal and lifestyle covariates are needed to clarify the mechanisms underlying sex–age interactions.

Our findings demonstrated that eosinophil-related inflammatory indices, ELR and EA-SIRI, were significantly associated with AD phenotype, suggesting a possible link between eosinophilic immune activity and lipid metabolism and vascular risk. This observation is in line with emerging evidence that eosinophils have been implicated in cardiovascular disease (CVD) and atherosclerosis beyond their classical roles in allergy and parasitic defence [53]. A recent study in the European Heart Journal provided mechanistic insight by demonstrating that eosinophil-derived cationic proteins can bind to bone morphogenetic protein receptors on vascular smooth muscle cells, potentially activating osteogenic pathways and promoting vascular calcification and atherogenesis [54]. Subclinical vascular changes have also been reported in asthmatic adults with elevated eosinophil counts, including reduced carotid strain, a marker of early arterial stiffness [55]. Another study found that eosinophil percentages in circulating leukocytes were independently associated with coronary artery disease severity and acute myocardial infarction [56]. Although these studies focused on eosinophil count, our findings extend this evidence by highlighting the relevance of eosinophil-based inflammatory indices, which integrate eosinophil activity with broader immune cell dynamics. These indices may better capture low-grade inflammation in atherogenic dyslipidemia, suggesting a role for immunometabolic crosstalk in lipid and cardiovascular risk.

Blood eosinophil count is a recognized marker of type 2 inflammation, particularly in asthma and allergic rhinitis, but the role of eosinophil-related composite indices in metabolic disorders remains less clear [57]. Emerging evidence suggests possible interactions between lipid metabolism, immune activation, and type 2 inflammation, with dyslipidemia potentially linked to Th2 polarization and chronic inflammation [58,59,60]. The coexistence of obesity and dyslipidemia in eosinophilic conditions such as nasal polyps and asthma further supports a potential shared immunometabolic basis [61,62,63]. A U.S. outpatient study also reported reduced rates of chronic rhinosinusitis among statin users, raising the possibility that lipid-lowering therapy may reduce eosinophil-related inflammation [64]. In our study, eosinophil-related indices (ELR and EA-SIRI) were elevated in individuals with atherogenic dyslipidemia, suggesting that metabolic disturbances may be associated with enhanced eosinophil-related inflammation. However, ROC analyses yielded modest AUCs, with Youden-derived cut-offs (ELR > 0.073 and EA-SIRI > 0.088) demonstrating only modest sensitivity and specificity. Thus, while these indices may reflect underlying immunometabolic processes, their current discriminatory capacity is limited, and the proposed thresholds should be considered exploratory and population-specific. Validation in larger, prospective cohorts will be essential to establish their reproducibility and clinical relevance.

Several limitations of this study should be acknowledged. Its cross-sectional design limits causal interpretation, and longitudinal studies are needed to assess the predictive utility of ELR and EA-SIRI. The absence of important covariates, including BMI, smoking status, and comorbidities, precluded multivariable adjustment, and residual confounding cannot be excluded. The lack of data on medication use could have further affected both lipid and inflammatory profiles. Another limitation is its single-center design and reliance on routine laboratory data from individuals seeking care or check-ups at Elta Medical Laboratories, which may introduce selection bias. Therefore, the study population may not fully represent the general population, and this potential bias should be considered when interpreting the findings. Furthermore, although the large sample size permitted subgroup and stratified analyses, this remains an exploratory study, and subgroup results should be interpreted with caution given the risk of multiple comparisons and limited statistical power. Despite these limitations, the study benefited from a large sample size, automated data collection that minimized measurement variability, and comprehensive lipid profile analysis, all of which strengthen the reliability of the findings.

## 5. Conclusions

Our findings highlight a significant association between eosinophil-related inflammatory indices and distinct dyslipidemia phenotypes, suggesting that eosinophil-driven inflammation may play a contributory role in the immunometabolic dysregulation underlying lipid abnormalities. Elevated levels of ELR and EA-SIRI were most evident in phenotypes characterized by hypertriglyceridemia and reduced HDL cholesterol, such as atherogenic and combined dyslipidemia. This distribution pattern supports the involvement of eosinophils in lipid metabolism and chronic low-grade systemic inflammation. As such, eosinophil-related indices may provide clinically meaningful insights into lipid-associated inflammatory states and serve as promising tools for cardiometabolic risk stratification.

## Figures and Tables

**Table 1:** Clinical Characteristics Across Dyslipidemia Phenotypes.

Variable	NLP	IHC	IHTG	ILHDL	AD	CD	*p*-Value
Age (yrs)	45.0 (37.0–53.0)	46.0 (38.0–54.0)	47 (39–55)	45.0 (37.0–54.0)	48.0 (38.0–56.0)	45.0 (37.0–53.0)	0.1035
Sex (Female%)	62.26%	60.73%	64.00%	59.97%	53.08%	55.68%	---
WBC (×10^9^/μL)	5.55 (4.48–6.87)	5.51 (4.48–6.82)	6.22 (4.89–7.48)	5.68 (4.58–7.01)	6.09 (4.99–7.67)	5.79 (4.71–7.26)	<0.001
NEU (×10^9^/μL)	2.55 (1.69–3.55)	2.48 (1.67–3.52)	2.96 (1.91–4.08)	2.54 (1.78–3.67)	2.74 (2.00–3.72)	3.04 (1.96–3.83)	<0.001
MON (×10^9^/μL)	0.44 (0.36–0.54)	0.43 (0.35–0.53)	0.46 (0.37–0.57)	0.47 (0.38–0.57)	0.50 (0.41–0.61)	0.49 (0.39–0.60)	<0.001
LYM (×10^9^/μL)	2.32 (1.91–2.77)	2.31 (1.92–2.75)	2.60 (2.12–2.94)	2.35 (1.94–2.79)	2.48 (2.06–3.14)	2.6 (2.16–3.10)	<0.001
EOS (×10^9^/μL)	0.15 (0.09–0.23)	0.16 (0.10–0.24)	0.18 (0.11–0.26)	0.19 (0.11–0.28)	0.21 (0.14–0.30)	0.20 (0.13–0.30)	<0.001
Hb (g/dL)	14.5 (13.3–15.6)	14.7 (13.6–15.8)	14.8 (13.7–15.8)	14.9 (13.8–15.8)	14.8 (13.8–15.8)	14.8 (13.6–15.8)	<0.001
RBC (×10^12^/L)	5.53 (5.12–5.94)	5.66 (5.23–6.09)	5.76 (5.24–6.20)	5.75 (5.37–6.20)	5.85 (5.51–6.30)	5.84 (5.36–6.30)	<0.001
PLT (×10^9^/L)	326 (276–384)	322 (271–376)	334 (281–387)	314 (266–374)	299 (248–349)	314 (268–368)	<0.001
CRP (mg/L)	1.10 (1.01–1.20)	1.08 (1.00–1.20)	1.07 (0.98–1.20)	1.10 (1.02–1.20)	1.07 (0.98–1.20)	1.07 (0.98–1.20)	<0.001
FBG (mg/dL)	100 (93–109)	105 (95–114)	100 (93–109)	105 (96–115)	103 (94–113)	100 (93–109)	<0.001

Values are presented as median (IQR). Abbreviations: NLP, normolipidemic profile; IHC, isolated hypercholesterolemia; IHTG, isolated hypertriglyceridemia; ILHDL, isolated low high-density lipoprotein cholesterol; AD, atherogenic dyslipidemia; CD, combined dyslipidemia; WBC, white blood cells; NEU, neutrophils; LYM, lymphocytes; MON, monocytes; EOS, eosinophils; Hb, hemoglobin; RBC, red blood cells; CRP, C-reactive protein; FBG, fasting blood glucose.

**Table 2:** Lipid Profiles and Distribution of Dyslipidemia Phenotypes Across ELR Tertiles.

Variable	T1	T2	T3	*p*-Value
**Lipid Parameters**				
TG (mg/dL)	83 (63–113)	90 (68–121)	94 (71–123)	<0.001
HDL-C (mg/dL)	50 (44–58)	49 (43–57)	47 (42–55)	<0.001
TC (mg/dL)	180 (161–205)	185 (164–208)	183 (161–207)	0.003
LDL-C (mg/dL)	113 (95–134)	117 (99–137)	117 (97–138)	< 0.001
**DLD Phenotypes (%)**				
NLP	52.91	47.05	43.86	–
IHC	28.95	32.23	30.76	–
IHTG	2.63	2.50	2.41	–
ILHDL	9.13	9.22	13.31	–
AD	1.85	3.40	3.83	–
CD	4.52	5.60	5.82	–

Values are presented as median (IQR). Abbreviations: ELR, eosinophil-to-lymphocyte ratio; TG, triglycerides; TC, total cholesterol; HDL-C, high-density lipoprotein cholesterol; LDL-C, low-density lipoprotein cholesterol; DLD, dyslipidemia; NLP, normolipidemic profile; IHC, isolated hypercholesterolemia; IHTG, isolated hypertriglyceridemia; ILHDL, isolated low high-density lipoprotein cholesterol; AD, atherogenic dyslipidemia; CD, combined dyslipidemia.

**Table 3:** Lipid Profiles and Distribution of Dyslipidemia Phenotypes Across EA-SIRI Tertiles.

Variable	T1	T2	T3	*p* Value
**Lipid Parameters**				
TG (mg/dL)	82 (63–110)	89 (68–118)	96 (73–127)	<0.001
HDL-C (mg/dL)	50 (44–59)	49 (43–56)	47 (42–55)	<0.001
TC (mg/dL)	183 (161–206)	183 (163–207)	182 (161–206)	0.515
LDL-C (mg/dL)	115 (96–135)	115 (98–137)	116 (97–137)	0.209
**DLD Phenotypes (%)**				
NLP	51.27	49.03	43.52	–
IHC	31.45	31.24	29.25	–
IHTG	2.24	2.33	2.97	–
ILHDL	8.79	9.91	12.97	–
AD	1.81	2.67	4.61	–
CD	4.44	4.83	6.68	–

Values are presented as median (IQR). Abbreviations: EA-SIRI, eosinophil-adjusted systemic inflammation response index; TG, triglycerides; TC, total cholesterol; HDL-C, high-density lipoprotein cholesterol; LDL-C, low-density lipoprotein cholesterol; DLD, dyslipidemia; NLP, normolipidemic profile; IHC, isolated hypercholesterolemia; IHTG, isolated hypertriglyceridemia; ILHDL, isolated low high-density lipoprotein cholesterol; AD, atherogenic dyslipidemia; CD, combined dyslipidemia.

**Table 4:** Dyslipidemia Phenotype Frequencies in Relation to ELR and EA-SIRI Levels.

DLD Phenotype	N—ELR (%)	H—ELR (%)	N—EA-SIRI (%)	H—EA-SIRI (%)
NLP	51.52	43.52	49.99	35.32
IHC	29.86	31.62	31.97	22.57
IHTG	2.57	2.44	2.25	2.22
ILHDL	9.02	12.44	8.97	9.81
AD	2.29	3.94	2.22	3.17
CD	4.73	6.03	4.60	4.86

Abbreviations: ELR, eosinophil-to-lymphocyte ratio; EA-SIRI, eosinophil-adjusted systemic inflammation response index; DLD, dyslipidemia; NLP, normolipidemic profile; IHC, isolated hypercholesterolemia; IHTG, isolated hypertriglyceridemia; ILHDL, isolated low high-density lipoprotein cholesterol; AD, atherogenic dyslipidemia; CD, combined dyslipidemia.

**Table 5:** Risk Estimates of DLD Phenotypes Based on Elevated ELR and EA-SIR.

	ELR			EA-SIR		
DLD Phenotype	OR	95% CI	*p*-Value	OR	95% CI	*p*-Value
IHC	0.999	0.89–1.12	*p* = 0.986	1.25	1.12–1.40	*p* < 0.001
IHTG	1.40	1.03–1.90	*p* = 0.030	1.12	0.82–1.52	*p* = 0.467
ILHDL	1.55	1.32–1.82	*p* < 0.001	1.63	1.39–1.92	*p* < 0.001
AD	2.02	1.52–2.68	*p* < 0.001	2.04	1.54–2.71	*p* < 0.001
CD	1.49	1.20–1.85	*p* < 0.001	1.51	1.22–1.87	*p* < 0.001

Abbreviations: ELR, eosinophil-to-lymphocyte ratio; EA-SIRI, eosinophil-adjusted systemic inflammation response index; DLD, dyslipidemia; NLP, normolipidemic profile; IHC, isolated hypercholesterolemia; IHTG, isolated hypertriglyceridemia; ILHDL, isolated low high-density lipoprotein cholesterol; AD, atherogenic dyslipidemia; CD, combined dyslipidemia.

**Table 6:** Diagnostic Performance of ELR and EA-SIRI for Predicting Dyslipidemia Phenotypes.

	ELR		EA-SIRI	
DLD Phenotype	AUC (95% CI)	*p*-Value	AUC (95% CI)	*p*-Value
IHC	0.53 (0.51–0.55)	<0.001	0.51 (0.49–0.52)	0.340
IHTG	0.52 (0.48–0.57)	0.309	0.56 (0.52–0.61)	0.005
ILHDL	0.57 (0.55–0.60)	<0.001	0.57 (0.55–0.60)	<0.001
AD	0.60 (0.56–0.64)	<0.001	0.62 (0.58–0.66)	<0.001
CD	0.57 (0.53–0.61)	<0.001	0.60 (0.57–0.64)	<0.001

Abbreviations: ELR, eosinophil-to-lymphocyte ratio; EA-SIRI, eosinophil-adjusted systemic inflammation response index; DLD, dyslipidemia; IHC, isolated hypercholesterolemia; IHTG, isolated hypertriglyceridemia; ILHDL, isolated low high-density lipoprotein cholesterol; AD, atherogenic dyslipidemia; CD, combined dyslipidemia.

**Table 7:** Cut-off values of ELR and EA-SIRI for predicting dyslipidemia.

Parameter	Criterion	Cut-Off Value	Sensitivity % (95% CI)	Specificity % (95% CI)
ELR	Youden (balanced)	>0.073	58.8 (52.0–65.2)	58.7 (57.0–60.4)
	Rule-out (high sensitivity)	>0.037	90.5 (85.8–93.8)	19.6 (18.3–21.0)
	Rule-in (high specificity)	>0.159	10.4 (7.0–15.3)	91.5 (90.5–92.4)
EA-SIRI	Youden (balanced)	>0.088	58.8 (52.0–65.2)	58.6 (56.9–60.3)
	Rule-out (high sensitivity)	>0.030	90.1 (85.3–93.4)	20.0 (18.6–21.3)
	Rule-in (high specificity)	>0.234	16.1 (11.8–21.7)	90.0 (89.0–91.0)

Abbreviations: ELR, eosinophil-to-lymphocyte ratio; EA-SIRI, eosinophil-adjusted systemic inflammation response index.

## Data Availability

Data are available from the corresponding author, Y.A., upon reasonable request and with permission of Elta Medical Laboratory.

## Supplementary Materials

The following supporting information can be downloaded at: https://www.mdpi.com/article/10.3390/medicina61091579/s1, Table S1: Comparative findings of eosinophil-related indices and lipid abnormalities across different cohorts.
