# Supplementary material for: Leukocyte-Based Inflammatory Profiles Across Dyslipidemia Phenotypes: Patterns of Eosinophil-Related Indices
Source: Medicina (Kaunas). 2025 Aug 31;61(9):1579. doi: 10.3390/medicina61091579 (PMC12471707; doi:10.3390/medicina61091579)
Supplement: Supplementary file 1 [file medicina-61-01579-s001.zip › medicina-3821136-supplementary.pdf]

Table S1: Comparative findings of eosinophil-related indices and lipid abnormalities across different cohorts.

Study	Population	Sample Size	Marker(s)	Lipid Outcomes	Key Notes
Netherlands cohort [33]	General population	~8,000	Eosinophil count	HDL-C, LDL-C, TG, TC	eosinophilia linked to adverse lipid profile
Japanese cohort [34]	Community-based	~6,000	Eosinophil count	TC, TG	supports link with metabolic dysregulation
U.S. Multi-Ethnic Study of Atherosclerosis (MESA) [35]	Multi-ethnic adults (White, Black, Hispanic, Chinese)	~6,500	Eosinophil count	Lipid fractions	No significant eosinophil–lipid associations
British cohort [36]	UK population	~5,000	Eosinophil %	LDL-C, TG	Higher eosinophils linked to more favorable lipid profile
Switzerland subjects [40]	Murine and human adipose tissue	NA	Eosinophils in adipose	Metabolic markers	Showed eosinophil loss contributes to metabolic deterioration
Denmark subjects [48]	Human vascular samples	Experimental	Eosinophil proteins	Vascular calcification pathways	Mechanistic; ECP binding to BMP receptors
Current study	Saudi adults	~7,000	ELR, EA-SIRI	Dyslipidemia phenotypes (esp. AD, combined)	Strongest in high TG/low HDL phenotypes; sex/age-specific patterns
